# Mean Duration of Mechanical Ventilation among Newborns Admitted to the Neonatal Intensive Care Unit in a Tertiary Care Centre: A Descriptive Cross-sectional Study

**DOI:** 10.31729/jnma.8201

**Published:** 2023-06-30

**Authors:** Ruby Thakur, Atindra Mishra

**Affiliations:** 1 Department of Paediatrics, Bajrabarahi Chapagaun Hospital, Bajrabarahi, Lalitpur, Nepal; 2 Department of Paediatrics, National Medical College, Bhediyahi, Birgunj, Nepal

**Keywords:** *mechanical ventilation*, *neonatal death*, *neonatal intensive care unit*, *neonate*

## Abstract

**Introduction:**

Neonatal deaths account for the majority of deaths in the first year of life. Life-threatening apnoea or cardiovascular collapse needs cardiopulmonary resuscitation. Technological advancements, such as the administration of antepartum steroids, replacement of surfactants, nasal continuous positive airway pressure, and mechanical ventilation have led to improved neonatal survival, especially for premature neonates. The aim of this study was to find out the mean duration of mechanical ventilation among newborns admitted to the Neonatal Intensive Care Unit in a tertiary care centre.

**Methods:**

A descriptive cross-sectional study was conducted from 15 November 2020 to 15 April 2021 in a tertiary care centre. Ethical approval was taken from Institutional Review Committee (Reference number: F-NMC|504|076|077). The neonates of gestational age between 27-37 weeks of gestation admitted to the Neonatal care unit were included in the study. Neonates with birth weight <500 g and those babies having major, surgically uncorrectable lethal anomalies were excluded from the study. Convenience sampling method was used. Point estimate and 95% Confidence Interval were calculated.

**Results:**

Among 86 neonates, the mean duration of mechanical ventilation was 2.19±1.42 days. The mean weight of neonates was 2.63±0.81 kg. A total of 71 (82.56%) were male and 15 (17.44%) were female.

**Conclusions:**

The mean duration of mechanical ventilation among newborns admitted to the neonatal intensive care unit in a tertiary care centre was lower than in other studies conducted in similar settings.

## INTRODUCTION

Neonatal deaths account for more than two-thirds of all deaths in the first year of life and about half of all deaths in children under five. According to the Nepal Demographic and health survey 2016 (NDHS 2016), neonatal mortality has decreased from 33 per 1000 live births to 21 per 1000.^1^

Critically sick neonates, who develop life-threatening apnoea or cardiovascular collapse, need cardiopulmonary resuscitation. Infants with progressive respiratory distress with impending respiratory failure or tiring respiratory muscles can be supported and saved by assisted ventilation facilities.^2^ Technologic advancements, such as the administration of antepartum steroids, replacement of surfactant, nasal continuous positive airway pressure (nCPAP), and mechanical ventilation have led to improved neonatal survival, especially for premature neonates.^3^ Improving intensive care facilities for neonates in the country could be one of the effective interventions to achieve the global target of reducing under-five mortality by two thirds.^2^

The aim of this study was to find out the mean duration of mechanical ventilation among newborns admitted to the Neonatal Intensive Care Unit in a tertiary care centre.

## METHODS

This descriptive cross-sectional study was conducted from 15 January 2021 to 15 April 2021 at the neonatal intensive care unit of National Medical College, Bhediyahi, Birgunj, Nepal after obtaining ethical approval from the Institutional Review Committee (Reference number: F-NMC|504|076|077). The neonates of gestational age between 27-37 weeks of gestation admitted to the Neonatal care unit were included in the study. Neonates with birth weight <500 g and those babies having major, surgically uncorrectable lethal anomalies were excluded from the study. Convenience sampling method was used. The sample size was calculated using the following formula:


$$\begin{array}{ll} \text{n} & ={\text{Z}}^{2}\times \frac{{\text{σ}}^{\text{2}}}{{\text{e}}^{\text{2}}}\\ & =1.96^{2}\times \frac{6.18^{2}}{2^{2}}\\ & =36 \end{array}$$

Where,

n = minimum required sample size
Z = 1.96 at 95% Confidence Interval (CI)
p = standard deviation is taken as from published literature^4^
q = 1-p
e = margin of error, 1%

The minimum required sample size was 36. However, the final sample size taken was 86. The available demographic and clinical parameters were recorded as per the proforma.

Data were entered and analysed using Microsoft Excel 2016. Point estimate and 95% Confidence Interval were calculated.

## RESULTS

Among 86 neonates, the mean duration of mechanical ventilation was 2.19±1.42 days. The mean weight of neonates was 2.63±0.81 kg. The majority 71 (82.56%) were male and 15 (17.44%) were female. The mortality was 28 (32.56%) (Table 1).

**Table 1:** Underlying Conditions (n= 86).

Parameters	n (%)
Perinatal asphyxia	45 (52.32)
Hypoxic ischemic encephalopathy	42 (48.84)
Shock	42 (48.84)
Sepsis	34 (39.53)
Meconium aspiration syndrome	13 (15.12)
Low birth weight	5 (5.81)
Congestive cardiac failure	5 (5.81)
Preterm	4 (10.46)
Disseminated intravascular coagulation	2 (2.32)
Myocarditis	2 (2.32)
ABO incompatibility	2 (2.32)
Pneumothorax	1 (1.16)
Imperforate anus	1 (1.16)
Necrotizing enterocolitis	1 (1.16)
Small for gestational age	1 (1.16)
Pulmonary haemorrhage	1 (1.16)
Gastroschisis	1 (1.16)
Intestinal obstruction	1 (1.16)
Atrial septal defect	1 (1.16)
Intracranial bleed	1 (1.16)
Very low birth weight	1 (1.16)
Congenital heart disease	1 (1.16)

## DISCUSSION

In this study, the mean duration of mechanical ventilation was 2.19±1.42 days which is lower than the other similar study.^5^ In our study total of 71 (82.56%) were male and 15 (17.44%) were female, and other similar studies found that 78 (55.3%) were male and 63 (44.7%) were female.^5^ The mean weight of neonates was 2.63±0.81 kg in our study. Similar to our study, the mean birth weight was found to be 2779.37±827.06 g in another study,^5^ and weight was 2000±690 g in another similar study.^6^

In our study a total of 86 neonates were ventilated. Similar to our study, other studies conducted found that out of 50 neonates were ventilated in the study,^6^ similarly 6.83% of neonates required mechanical ventilation in another similar study.^7^ In addition to this another study found 30.7% of neonates were ventilated,^8^ and another study found 72 neonates required mechanical ventilation.^9^ Our study has found that 52.32% of the mechanically ventilated newborn has underlying perinatal asphyxia however similar study found that RDS (58.9%) was the most common indication for mechanical ventilation.^5^ In our study, hypoxic-ischemic encephalopathy was present in 48.84% of neonates which is lower in comparison to another study in which prevalence was 20.2%.^8^

In our study, asphyxia was present in 45 (52.32%) which is higher than the other similar study in which asphyxia was present in 24%.^6^ Pneumothorax was present in 1 (1.16%) another similar study found the prevalence to be 4.96%.^5^ In our study, the prevalence of hypoxic-ischemic encephalopathy was 42 (48.84%), which is higher than a similar study in which the prevalence was 12.5%.^9^ In our study congenital heart disease was present in 1 (1.16%) similarly in another study it was about 4.96%.^5^ Meconium aspiration syndrome was present in 13 (15.12%), which is lower than one of the similar studies.^9^ In our study, mortality was 28 (32.56%) similar to this the mortality was found to be 48.2% in a study.^5^

The limitation of this study is that since it is a singlecentered descriptive cross-sectional study, the result cannot be generalised and the outcomes could not be analyzed with analytical parameters.

## CONCLUSIONS

The mean duration of mechanical ventilation among newborns admitted to the neonatal intensive care unit in a tertiary care centre was found to be lower than in other studies conducted in similar settings.
